# Treatment of Menstrual Disorders

**Published:** 1904-08

**Authors:** 


### Treatment of Menstrual Disorders.

Willey, Louisville, (*South. Practitioner*) says the term amenorrhea which is used to mean the total absence of the menstrual discharge, or a marked deficiency in the quantity of the flow, may be physiological or pathological. During pregnancy the absence of the menstrual discharge is physiological and is not here considered. When pathological, the cause of amenorrhea may be said in general to be: (1) taking cold, at or near the menstrual epoch; (2) severe mental perturbation, as fright, sorrow, or great elation of spirit; (3) it may be symptomatic in several affections, as tuberculosis, anemia, chlorosis, syphilis, typhoid fever, nephritis, pelvic peritonitis, and other morbid conditions; (4) obesity; (5) luxurious life, or overtaxing the nervous system; (6) stenosis or atresia of the cervical canal, or imperfect development of the tubes, ovaries or uterus; (7) vicarious menstruation may make the condition obscure, there being a discharge at the regular monthly periods from the nose, lungs, bladder, stomach, nipple, or other part.

Treatment should be addressed to removing the cause. When the amenorrhea is caused by having contracted cold, the patient should have a warm sitz bath, and hot applications should be applied to the abdomen and thighs. Often a hot vaginal injection will serve a most useful purpose, and a laxative, perferably a saline, will greatly aid in bringing on the flow.

In amenorrhea, delayed menstruation and dysmenorrhea, ergoapiol has acted in his hands in a most satisfactory manner. In scanty menstruation he found it particularly valuable and cites the following case:

A lady, aged 33, had scanty menstruation which had covered the period of a year. At no time in the year had her menstrual period been longer than eighteen hours, but generally twelve hours told the tale. Her menses were not only scanty, but the color of the menstrual blood was pale, and the odor disagreeable. This woman had no associated disease that most searching examination could bring out. Still she had steadily increased in flesh for the last two years, and to this I attributed the amenorrhea.

This patient was put upon systematic exercise and a dietary that was rational, and ergoapiol (Smith) was administered with regularity, a capsule four times a day. After two months her menstruation became normal and the remedy was discontinued.

Another case cited is as follows:

A girl 20 years old was sent to Willey by the matron of a boarding school. She enjoyed good health prior to entering the school, but for three months had not menstruated, and was suffering constantly with vertigo and had attacks of hysteria. Attributing the amenorrhea to change of conditions of life—that of an open life on a farm to that of a shut-in inactive life, ergoapiol (Smith) was given after each meal for two weeks prior to the day of her usual menstruation. Her menses soon appeared and she has since had no further trouble.

In the course of the article the author cites a number of cases and says:

"My experience with ergoapiol (Smith) is such that I regard it as an indispensable remedy in all expressions of amenorrhea along with proper remedies for any diseased condition associated in the causation of the affection. Of course those cases where the amenorrhea is due to atresia of the cervical canal, and to any other condition which is remedial only by surgical means, drugs will prove of no avail. The same can be said of instances in the amenorrhea due to a rudimentary state of the female organs of reproduction."

